# The Curcumin Analogue, EF-24, Triggers p38 MAPK-Mediated Apoptotic Cell Death via Inducing PP2A-Modulated ERK Deactivation in Human Acute Myeloid Leukemia Cells

**DOI:** 10.3390/cancers12082163

**Published:** 2020-08-04

**Authors:** Pei-Ching Hsiao, Jer-Hwa Chang, Wei-Jiunn Lee, Chia-Chi Ku, Meng-Ying Tsai, Shun-Fa Yang, Ming-Hsien Chien

**Affiliations:** 1 School of Medicine, Chung Shan Medical University, Taichung 402, Taiwan; cshy046@csh.org.tw; 2 Department of Internal Medicine, Chung Shan Medical University Hospital, Taichung 402, Taiwan; 3 School of Respiratory Therapy, College of Medicine, Taipei Medical University, Taipei 110, Taiwan; m102094030@tmu.edu.tw; 4 Division of Pulmonary Medicine, Department of Internal Medicine, Wan Fang Hospital, Taipei Medical University, Taipei 116, Taiwan; 5 Pulmonary Research Center, Wan Fang Hospital, Taipei Medical University, Taipei 116, Taiwan; 6 Department of Urology, School of Medicine, College of Medicine, Taipei Medical University, Taipei 110, Taiwan; lwj5905@gmail.com; 7 Department of Medical Education and Research, Wan Fang Hospital, Taipei Medical University, Taipei 116, Taiwan; 8 Graduate Institute of Clinical Medicine, College of Medicine, Taipei Medical University, Taipei 110, Taiwan; laboyku@gmail.com; 9 Institute of Medicine, Chung Shan Medical University, Taichung 402, Taiwan; vickyfatfat5252@gmail.com; 10 Department of Medical Research, Chung Shan Medical University Hospital, Taichung 402, Taiwan; 11 TMU Research Center of Cancer Translational Medicine, Taipei Medical University, Taipei 110, Taiwan; 12 Traditional Herbal Medicine Research Center, Taipei Medical University Hospital, Taipei 110, Taiwan

**Keywords:** acute myeloid leukemia, apoptosis, EF-24, protein phosphatase 2 a, p38 mitogen-activated protein kinase, extracellular-regulated protein kinase

## Abstract

Curcumin (CUR) has a range of therapeutic benefits against cancers, but its poor solubility and low bioavailability limit its clinical use. Demethoxycurcumin (DMC) and diphenyl difluoroketone (EF-24) are natural and synthetic curcumin analogues, respectively, with better solubilities and higher anti-carcinogenic activities in various solid tumors than CUR. However, the efficacy of these analogues against non-solid tumors, particularly in acute myeloid leukemia (AML), has not been fully investigated. Herein, we observed that both DMC and EF-24 significantly decrease the proportion of viable AML cells including HL-60, U937, and MV4-11, harboring different NRAS and Fms-like tyrosine kinase 3 (FLT3) statuses, and that EF-24 has a lower half maximal inhibitory concentration (IC_50_) than DMC. We found that EF-24 treatment induces several features of apoptosis, including an increase in the sub-G_1_ population, phosphatidylserine (PS) externalization, and significant activation of extrinsic proapoptotic signaling such as caspase-8 and -3 activation. Mechanistically, p38 mitogen-activated protein kinase (MAPK) activation is critical for EF-24-triggered apoptosis via activating protein phosphatase 2A (PP2A) to attenuate extracellular-regulated protein kinase (ERK) activities in HL-60 AML cells. In the clinic, patients with AML expressing high level of PP2A have the most favorable prognoses compared to various solid tumors. Taken together, our results indicate that EF-24 is a potential therapeutic agent for treating AML, especially for cancer types that lose the function of the PP2A tumor suppressor.

## 1. Introduction

Acute myeloid leukemia (AML) is the most common acute form of leukemia in adults, characterized by the overproduction of malignant progenitor myeloid cells in bone marrow and the peripheral bloodstream. Nowadays, the primary treatment for AML is chemotherapy [1], but chemoresistance and undesirable drug-derived side effects have prompted research into a range of other alternatives for AML treatment such as natural products harboring lower toxicity [2].

Curcumin (CUR), a polyphenolic natural product derived from turmeric, exerts multiple therapeutic effects on various diseases [3,4]. Hundreds of clinical trials have been performed to evaluate its efficacy in treating various diseases including cancer [5,6], which reported that CUR is safe and well tolerated in patients, even at high doses. However, few of those clinical trials showed positive outcomes [7], mainly due to its low solubility and poor bioavailability (<1%) [8]. Demethoxycurcumin (DMC) and diphenyl difluoroketone (EF-24) are natural and synthetic CUR analogues, respectively, that display multiple potent bioactivities and increased bioavailability compared to CUR [9,10]. For example, EF-24 was reported to have higher oral bioavailability (60%) and to be much safer than a chemotherapeutic drug in mice [11,12]. In addition, EF-24 exhibited a 10~20-fold lower 50% growth inhibitory concentration (IC_50_) than CUR in various solid tumor cells including ovarian, cervical, lung, breast, and prostate cancer cells [13,14,15,16]. Although these two CUR analogues, EF-24 and DMC, were reported to inhibit the proliferation of various solid tumor cells in in vitro and in vivo models [9,10], the precise impacts of these analogues on non-solid tumors, particularly AML, are still unclear.

The serine/threonine protein phosphatase protein phosphatase 2A (PP2A) comprises scaffold A, regulatory B, and catalytic C subunits in mammalian cells, and is responsible for the inactivation or negative regulation of numerous signaling pathways correlated with tumorigenesis. For example, phosphatidylinositol 3-kinase (PI3K)/Akt and its downstream signaling mammalian target of rapamycin (mTOR), p70S6K, and mitogen-activated protein kinase (MAPK) pathways mean that these are the main pathways affected by PP2A. In addition, PP2A was reported to target components involved in Wnt (glycogen synthase kinase (GSK)-3β, β-catenin) signaling [17], apoptosis (Bad, FOXO) [18], and cell cycle regulation (cdc25, WEE1, pRb) [19]. Many human cancers are associated with PP2A dysfunction, and PP2A is recognized as a druggable tumor suppressor for various cancer types including AML [20,21]. For example, Smith et al. observed low PP2A activity in leukemic blasts from AML patients, and pharmacological activation of PP2A can enhance the cytotoxicity of fms-like tyrosine kinase 3 (FLT3) tyrosine kinase inhibitors (TKIs) in FLT3^+^ AML cells via targeting extracellular-regulated protein kinase (ERK) and Akt [22]. In addition, a PP2A activator was shown to display synergy with Ara-C in NRAS-mutant AML cells [23]. Recently, CUR was reported to exert anticancer potential via inducing or downregulating PP2A activities in different solid tumor types [24,25], but the role of PP2A in the antileukemic effect of CUR analogues still requires elucidation.

In the current study, we first evaluated the cytotoxic effects of DMC and EF-24 in HL-60, U937, and MV4-11 AML cell lines, which harbor the wild-type (WT) or mutant form of FLT3 and NRAS (FLT3-WT or FLT3-internal tandem duplication (ITD) and NRAS-WT or NRAS Q61L mutation). Our results showed that both DMC and EF-24 decrease the proportion of viable cells in these AML cell lines, and EF-24 showed a more-potent antileukemic effect than DMC. Mechanistically, we found that EF-24 induces p38 activation, which negatively regulates ERK activity in a PP2A-dependent manner, and further induces caspase-mediated apoptosis of HL-60 AML cells.

## 2. Results

### 2.1. EF-24 Exerts a More Potent Effect than DMC in Decreasing the Proportion of Viable Cells in AML Cell Lines Harboring Different FLT3 and NRAS Statuses

**Figure 1:**
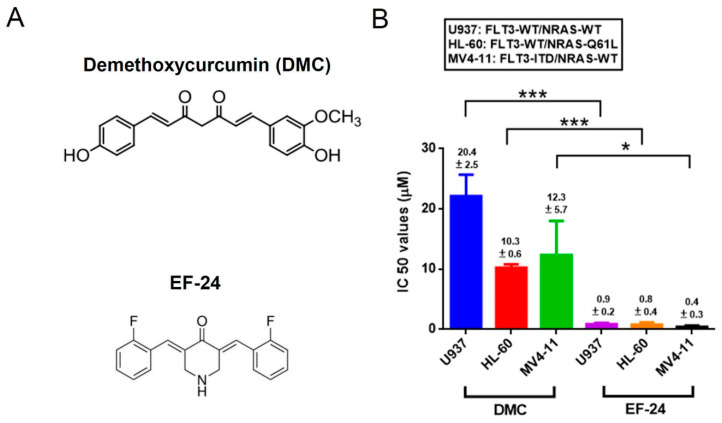
Comparison of cytotoxic effects of demethoxycurcumin (DMC) and EF-24 in human acute myeloid leukemia (AML) cells harboring different Fms-like tyrosine kinase 3 (FLT3) and NRAS statuses. (**A**) The chemical structures of DMC and EF-24 are shown in upper and lower panels, respectively. (**B**) The human HL-60, U937, and MV4-11 AML cell lines were treated with different concentrations of DMC (0, 0.125, 0.25, 1, 2, 4, 8, 16, and 32 µM) or EF-24 (0, 0.125, 0.25, 1, 2, and 4 µM) in complete medium for 24 h. A CCK-8 assay was used to determine the proportion of viable cells, which was calculated relative to control. Half-maximal inhibitory concentration (IC_50_) values derived from the results of the CCK-8 assay are the average of three separate experiments. * *p* < 0.05, *** *p* < 0.001 vs. IC_50_ value of DMC.

The chemical structures of DMC and EF-24 are shown in Figure 1A. To first evaluate the antileukemic potency of DMC compared to EF-24, we conducted a cell cytotoxicity test with a CCK-8 assay on a panel of AML cells, which represented different FLT3 and NRAS statuses (HL-60: FLT3-WT/NRAS-Q61L; U937: FLT3-WT/NRAS-WT; and MV4-11: FLT3-ITD/NRAS-WT). Treatment of cells with either EF-24 or DMC for 24 h significantly decreased the proportion of viable cells. We observed that the half-maximal (50%) inhibitory concentration (IC_50_) values of EF-24 and DMC were in the range of 0.4~0.9 and 10.3~20.4 µM, respectively, for these AML cell lines, as summarized in Figure 1B. The results suggested that EF-24 is effective against all AML cell lines, and the IC_50_ value was significantly at least 10-fold lower than that of DMC. Therefore, we chose EF-24 for subsequent experiments.

### 2.2. EF-24 Treatment Results in Extrinsic Apoptotic Cell Death of AML Cells

**Figure 2:**
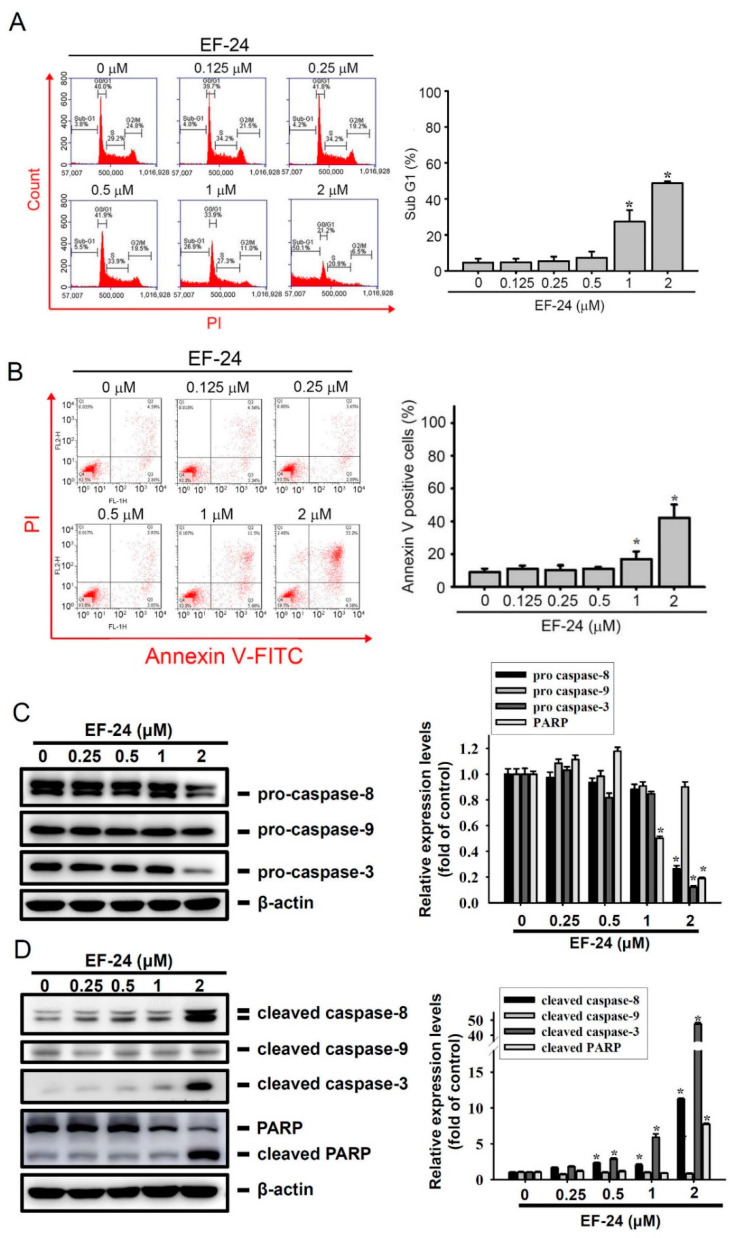
Effects of the distribution of cell-cycle phase and apoptosis in EF-24-treated human acute myeloid leukemia (AML) cells. (**A**) HL-60 cells were treated with different concentrations of EF-24 (0∼2 µM) for 24 h. The distribution of cell-cycle phases and sub-G_1_ phase (apoptosis) were analyzed by FACS after propidium iodide (PI) staining. Left panel, a representative example; right panel, the percentage of cell population distributed in the sub-G_1_ phase (*n* = 3). (**B**) An annexin-V and PI double-staining flow cytometry was used to quantify apoptotic cells in HL-60 cells treated with EF-24 (0~2 μM) for 24 h. Left panel, a representative example. In this dot plot, cells in early apoptosis (Annexin-V^+^/PI^−^) and late apoptosis (Annexin-V^+^/PI^+^) are shown in the bottom right quadrant and top right quadrant, respectively. Data are expressed as the mean ± standard deviation (SD) of three independent experiments. * *p* < 0.05 compared to the vehicle group. (**C**,**D**, left panel) HL-60 cells were treated with indicated concentrations of EF-24 for 24 h, and the protein levels of pro- and cleaved caspases-3, -8, and -9, and poly(ADP-ribose) polymerase (PARP) were determined by a Western blot analysis (**C**,**D**, right panel). The quantitative results of these protein levels were adjusted by β-actin protein levels, and results are expressed as multiples of induction beyond each respective control. Values are presented as the mean ± SD from three independent experiments. * *p* < 0.05 vs. vehicle group. Whole Blots for Western Blot analysis for Figure 2C are shown in the Figure S9. Whole Blots for Western Blot analysis for Figure 2D are shown in the Figure S10.

To investigate how EF-24 can attenuate the number of viable AML cells, we first performed flow cytometry to determine the effect of EF-24 on the distribution of cell-cycle and sub-G1 phases in HL-60 AML cells (Figure 2A, left panel). The right panel of Figure 2A shows that the sub-G_1_ apoptotic fraction was slightly and dramatically increased in HL-60 cells treated with 1 and 2 μM EF-24, respectively (Figure 2A, right panel). Apoptosis triggered by EF-24 was further confirmed by detecting another hallmark of apoptosis, translocated phosphatidylserine (PS), using Annexin V-FITC/propidium iodide (PI) double-staining. Figure 2B showed that the proportion of early and late apoptotic cells all dramatically increased after treating HL-60 cells with 2 µM EF-24. In addition to HL-60 cells, increases in the sub-G_1_ apoptotic fraction (Figure S1) and translocation of PS (Figure S2) were also observed in U937 cells. These findings indicated that EF-24 can trigger apoptotic cell death in AML cells. To investigate the underlying mechanism of EF-24-induced apoptosis, activation of the initiator of an intrinsic pathway (caspase-9), an extrinsic pathway (caspase-8), and the final executioner (caspase-3) was detected in HL-60 AML cells. The results showed that EF-24 (0.25~2 μM for 24 h) concentration-dependently induced the degradation of procaspases-8 and -3 and upregulation of active caspases-8 and -3, but had no effect on activation of caspase-9. Active caspase-3-mediated cleavage of poly (ADP ribose) polymerase (PARP) was also concentration-dependently induced by EF-24 treatment (Figure 2C,D). We observed that relative expressions of cleaved caspase-8, caspase-3, and PARP were higher in cells treated with 2 μM EF-24 compared to cells treated with 1 or 0.5 μM EF-24. In addition to HL-60 cells, EF-24 also concentration-dependently induced the degradation of procaspase-8 and activation of caspase-3 in MV4-11 cells (Figure S3). Taken together, these results indicated that the antileukemic effect induced by EF-24 is at least partly via the activation of an extrinsic apoptotic pathway. In addition to apoptosis induction by EF-24, we observed that EF-24 (0.125~2 μM) treatment for 24 h induced the accumulation of cells in the S phase compared to vehicle-treated HL-60 and U937 cells (Figure S4), suggesting that cell cycle arrest might also be involved in the antileukemic effects of EF-24.

### 2.3. EF-24-Induced Apoptosis of HL-60 AML Cells via Triggering p38 MAPK Activation

**Figure 3:**
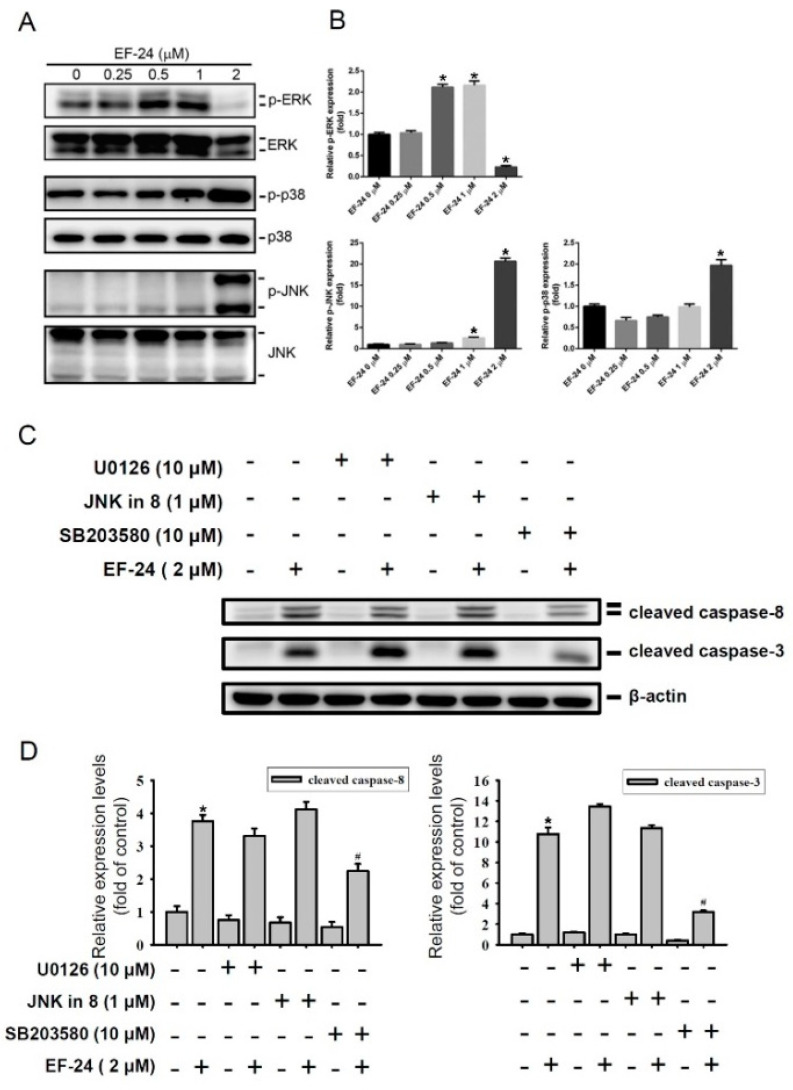
The p38 mitogen-activated protein kinase (MAPK) signaling is essential for EF-24-induced caspase-mediated apoptosis. (**A**,**B**) After 24 h treatment of HL-60 cells with indicated concentrations (0~2 µM) of EF-24, levels of phosphorylated extracellular signal-regulated kinase (ERK)1/2 (p-ERK1/2), p38 (p-p38), and c-Jun N-terminal kinase (JNK)1/2 (p-JNK1/2) were detected by a Western blot analysis (A). (B) Total ERK1/2, p38, and JNK1/2 protein levels were used to adjust quantitative results of p-ERK1/2, p-p38, and p-JNK1/2 protein levels, which are expressed as multiples of induction beyond each respective control. Values are presented as the mean ± SD of three independent experiments. * *p* < 0.05 vs. vehicle group. (**C**,**D**) After pretreatment of HL-60 cells with or without 10 µM U0126, 1 µM JNK-IN-8, or 10 µM SB203580 for 1 h, cells were further treated with EF-24 (2 µM) or the vehicle for another 24 h. Cleaved caspase-3 and -8 levels were determined by a Western blot analysis (**C**). Protein quantification was analyzed using Image-pro plus processing software, and the quantified cleaved caspase-3 and -8 protein levels were adjusted to the β-actin level and are expressed as multiples of induction beyond each respective control (**D**). Values represent the mean ± SD of three independent experiments. * *p* < 0.05 vs. vehicle group; ^#^ *p* < 0.05 vs. EF-24-treated group. Whole Blots for Western Blot analysis for Figure 3A are shown in the Figure S11. Whole Blots for Western Blot analysis for Figure 3C are shown in the Figure S12.

MAPK signaling pathways, including the c-Jun N-terminal kinase 1/2 (JNK1/2) and p38 MAPK, were reported to be involved in the caspase-mediated apoptotic effect triggered by CUR or DMC in various cancer types [26,27,28]. We therefore examined whether EF-24 could influence the activation of MAPKs, and observed the dynamic changes in MAPK activities in response to treatment with different concentrations of EF-24 (Figure 3A). The activity of ERK, but not JNK1/2 or p38 MAPK, dominantly increased after 0.5 and 1 µM EF-24 treatment for 24 h. In contrast, 2 µM EF-24 treatment resulted in a dramatic decrease in ERK activity, increases in p38 and JNK1/2 activities, and further induction of caspase-mediated apoptosis in HL-60 cells (Figure 2 and Figure 3B). Similar to HL-60 cells, we observed that ERK activation was induced by 0.25 and 0.5 µM EF-24 treatment for 24 h in MV4-11 cells; 1 µM EF-24 treatment induced a decrease in ERK activity accompanied by an increase in p38 activity and further induced caspase-mediated apoptosis (Figures S3 and S5). To further elucidate relationships among 2 μM EF-24-triggered activation of caspases and MAPKs, HL-60 cells were pretreated with an ERK inhibitor, U0126; a JNK inhibitor, JNK-IN-8; or a p38 inhibitor, SB203580, for 1 h, treated with EF-24 for another 24 h, and then we analyzed the expression of cleaved caspases by Western blotting (Figure 3C). The results showed that only blockage of p38 activation significantly reversed the EF-24-induced activation of caspases-8 and -3 (Figure 3D), suggesting that activation of p38 might play an upstream regulator in EF-24-induced caspase activation and cell death in HL-60 cells.

### 2.4. Relationship between p38 and ERK Activation in EF-24-Triggered Apoptosis of HL-60 Cells

**Figure 4:**
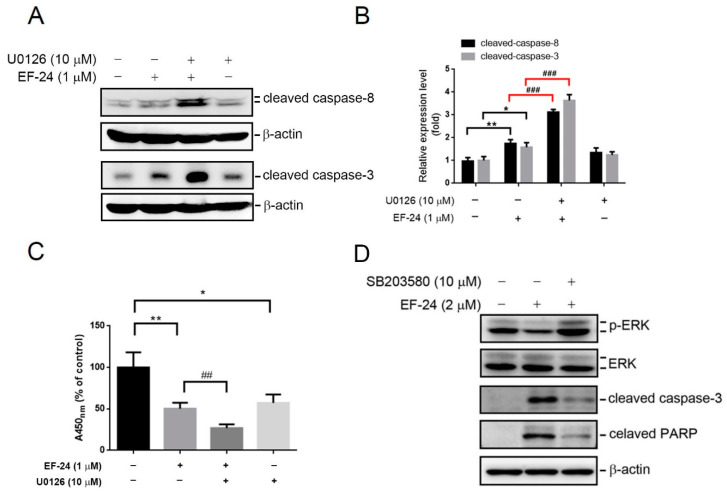
EF-24-induced p38 activation negatively regulates extracellular signal-regulated kinase (ERK) activity to induce apoptosis of HL-60 acute myeloid leukemia (AML) cells. (**A**–**C**) HL-60 cells were pretreated with or without 10 µM U0126 for 1 h followed by EF-24 (1 µM) or vehicle treatment for another 24 h. The Western blot analysis and CCK-8 assay were, respectively, used to determine the expression of cleaved caspase-8/-3 (**A**) and the proportion of viable cells (**C**). Levels of cleaved caspase-3 and -8 protein were quantified using Image-pro plus processing software and normalized to levels of β-actin (**B**). Values from CCK-8 assay are presented as the mean ± SD of three independent experiments. * *p* < 0.05, control vs. U0126 (10 µM); ** *p* < 0.01, control vs. EF-24 (1 µM); ^##^ *p* < 0.01, EF-24 (1 µM) vs. U0126 plus EF-24. (**D**) After pretreatment of HL-60 cells with or without 10 µM SB203580 for 1 h, cells were further treated with EF-24 (2 µM) or the vehicle for another 24 h. Levels of ERK, phosphorylated (p)-ERK, cleaved PARP, cleaved caspase-3, and β-actin were detected by a Western blot analysis. Whole Blots for Western Blot analysis for Figure 4A are shown in the Figure S13. Whole Blots for Western Blot analysis for Figure 4D are shown in the Figure S14.

In contrast to p38, ERK is correlated with the prosurvival function and drug resistance of leukemia cells [29,30]. Our results showed that moderate concentrations of EF-24 treatment (0.5 or 1 µM) for 24 h did not or only slightly triggered caspase-mediated apoptosis, but significantly induced ERK activation in HL-60 cells (Figure 2 and Figure 3A). These results suggested that ERK activation turned on by moderate concentrations of EF-24 may exert a prosurvival effect against the toxic effects of EF-24. We found that blocking of ERK activation by U0126 potentiated EF-24 (1 µM)-induced activation of caspases-8 and -3 (Figure 4A,B) in HL-60 cells. U0126 treatment also decreased the proportion of viable cells and further enhanced EF-24 (1 µM)-induced reduction of the proportion of viable cells (Figure 4C). The results from Figure 3A,B show that high-concentration EF-24 (2 µM) treatment simultaneously downregulated ERK activity and upregulated p38 activity. To further dissect the cross-talk between p38 activation and ERK deactivation in EF-24-mediated cell death, the results showed that pretreating HL-60 cells with SB203580 significantly reversed 2 µM EF-24-induced ERK deactivation and cleavage of caspase-3 and PARP (Figure 4D), suggesting that EF-24-induced p38 activation can negatively regulate ERK activity to trigger apoptosis.

### 2.5. EF-24-Induced p38 Activation Negatively Regulates ERK Activity in a PP2A-Dependent Manner

**Figure 5:**
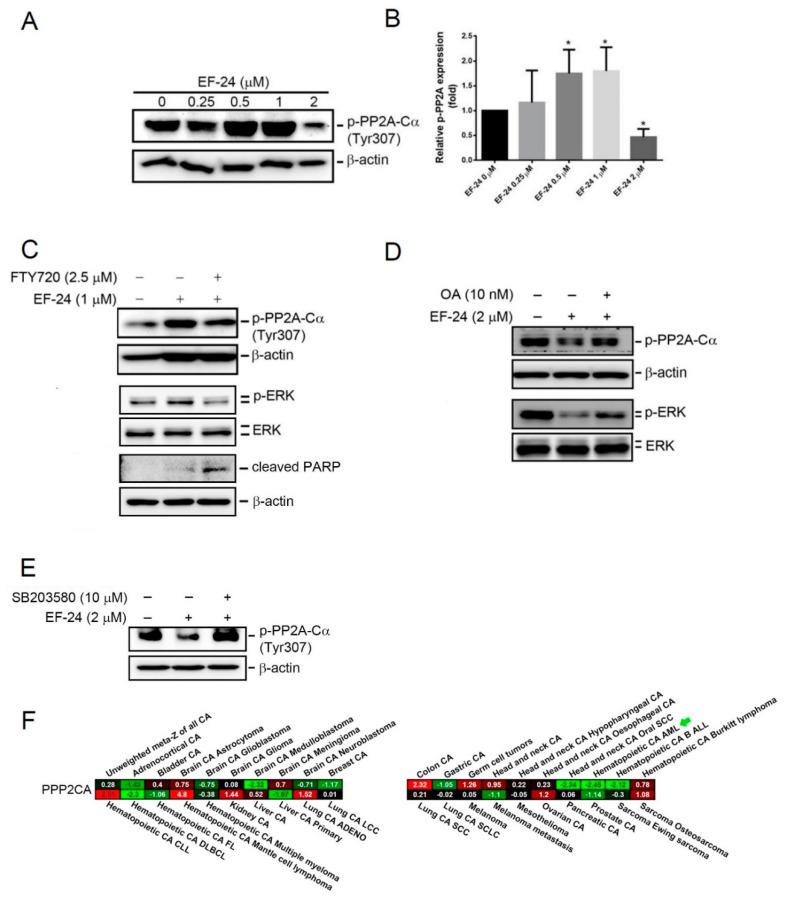
p38 is an upstream regulator of protein phosphatase 2A (PP2A)-modulated deactivation of extracellular signal-regulated kinase (ERK) in response to EF-24 treatment. (**A**) Treatment of HL-60 cells with indicated concentrations of EF-24 (0∼2 µM) for 24 h, and phosphorylation (p) levels of PP2A-Cα were determined by Western blot analysis. (**B**) Quantified p-PP2A-Cα protein levels were adjusted to β-actin levels and are expressed as multiples of induction beyond vehicle control. Data (mean ± SD) were from three independent experiments. * *p* < 0.05 vs. vehicle group. (**C**) Cotreatment of HL-60 cells with or without 2.5 µM FTY720 and EF-24 (1 µM) for 24 h. Expression of phosphorylated (p)-PP2A-Cα and p-ERK were determined by Western blot analysis. (**D**,**E**) HL-60 cells were pretreated with or without 10 nM okadaic acid (OA) (**D**) or 10 µM SB203580 (**E**) for 1 h followed by EF-24 (2 µM) treatment for another 24 h. A Western blot analysis was used to determine the expression levels of phosphorylated (p)-ERK and p-PP2A-Cα. (**F**) The pan-cancer expression of *PPP2CA* levels by meta-Z analysis from the PRECOG website. *PPP2CA* expression in cancer is associated with good survival in various cancer types. Whole Blots for Western Blot analysis for Figure 5A are shown in the Figure S15. Whole Blots for Western Blot analysis for Figure 5C are shown in the Figure S16. Whole Blots for Western Blot analysis for Figure 5D are shown in the Figure S17. Whole Blots for Western Blot analysis for Figure 5E are shown in the Figure S18.

Previous studies indicated that PP2A is an important serine/threonine phosphatase that regulates activities of MAPKs [31], and pharmacological activation of PP2A was reported to suppress FLT3-mediated growth of AML cells [22]. Phosphorylation of PP2A at Tyr 307 was reported to attenuate its enzyme activity [32]. Herein, we found that treatment with moderate (0.5 and 1 µM) and high (2 µM) concentrations of EF-24 with HL-60 AML cells increased and decreased the phosphorylation status of PP2A-Cα, respectively (Figure 5A,B), suggesting that declining and upregulated PP2A activities are dependent on different concentrations of EF-24 treatment. In addition, we also observed that PP2A activities were downregulated and upregulated by moderate (0.25 and 0.5 µM) and high (1 µM) concentrations of EF-24 treatment in MV4-11 cells, respectively (Figure S5). We noted that dynamic changes in PP2A activity were contrary to ERK activity in response to different concentrations of EF-24 treatment in HL-60 and MV4-11 AML cells (Figure 3B, Figure 5B, and Figure S5). Cotreatment with the PP2A activator, FTY720, reversed the 1 µM EF-24-induced PP2A deactivation and ERK activation and enhanced 1 µM EF-24-induced cleavage of PARP in HL-60 cells (Figure 5C). In contrast, we observed that pretreatment with the PP2A inhibitor, okadaic acid (OA), rescued 2 µM EF-24-induced PP2A activation as well as ERK deactivation (Figure 5D). Moreover, inhibition of PP2A by OA can also reverse the 1 µM EF-24-induced caspase-3 activation and PARP cleavage in MV4-11 cells (Figure S6). Taken together, these results suggested that a high concentration of EF-24 can induce an apoptotic effect in AML cells via inducing PP2A activity to negatively regulate ERK activity.

Due to the negative regulation of ERK activity by PP2A and p38 MAPK in response to high concentration of EF-24 treatment, we next investigated the crosstalk between PP2A and p38 MAPK activation induced by EF-24 in AML cells. Pretreatment of HL-60 cells with SB203580 significantly reversed EF-24-induced PP2A activation (Figure 5E), suggesting that p38 is an upstream regulator involved in EF-24-induced PP2A activation in AML cells. In the clinic, we used a pan-cancer prognostic database, PRECOG (PREdiction of Clinical Outcomes from Genomic profiles; https://precog.stanford.edu/index.php) [33], to analyze the prognostic values of PP2A-C. The results suggested that PP2A-C has a prognostic potential for favorable outcomes in a variety of solid and nonsolid tumors, and the most significantly prognostic effect of PP2A-C was observed in AML (Z score = −2.45, Figure 5F).

## 3. Discussion

AML is a hematological disorder pathologically defined by an abnormal increase in blasts with high mortality rates. Until now, conventional chemotherapy has had favorable outcomes; however, drug resistance to chemotherapy and disease recurrence usually occur within a short time, and several side effects have been observed [34,35]. Targeted therapies, such as the FLT3 inhibitor, have recently shown promising results, but the responses are still not durable [36]. There is considerable interest in therapy with drugs of plant origins because natural products have lower toxicity and fewer side effects [37]. For example, a natural product, arsenic trioxide (As_2_O_3_), is an effective and relatively safe drug in acute promyelocytic leukemia (APL) patients resistant to all-trans retinoic acid (ATRA) and conventional chemotherapy [38].

EF-24 is a monoketone analog of the natural product CUR, which was reported to exhibit higher potency than the chemotherapeutic drug cisplatin in inhibiting solid tumor cell growth [12]. In vitro, EF-24 significantly reduced proliferation of various solid cancer cells but showed non-cytotoxicity to normal cells. For example, Zou et al. reported that EF-24 inhibited survival of gastric cancer cell lines SGC-7901 and BGC-823 but did not affect the survival of normal human gastric epithelial cell line GES-1 or rat kidney proximal tubular epithelial cell line NRK-52E. The IC_50_ of EF-24 on SGC-7901 and BGC-823 cells are 3.1 and 5.1 μM, respectively, but the IC_50_ of EF-24 on GES-1 and NRK-52E cells are more than 10 μM [39]. In vivo, EF-24 showed high oral bioavailability and low toxicity in mice, but still inhibited the growth of human breast cancer in a mouse xenograft model [12].

Our current findings first revealed that EF-24 can attenuate the number of viable HL-60 FLT3-WT/NRAS (Q61L) AML cells through the induction of p38 MAPK-mediated apoptotic cell death via activating PP2A-mediated ERK deactivation. In addition to FLT3-WT AML cells, EF-24 decreased the proportion of viable MV4-11 FLT3-ITD/NRAS-WT AML cells via inducing caspase-mediated apoptosis. The IC_50_ values of EF-24 are in the range of 0.4~0.9 μM for these AML cells, but the cytotoxic effect of EF-24 on normal myeloid progenitors should be further investigated in the future. Inhibition of PP2A was recently observed in cancer cells downstream of aberrantly active oncogenic pathways driven by receptor tyrosine kinases (RTKs). In chronic lymphocytic leukemia (CLL) cells, LYN overexpression led to PP2A-C hyperphosphorylation and inactivation via increased association of PP2A-C with the PP2A inhibitor, SET [40]. In AML, Smith et al. reported oncogenic FLT3 activation could inactivate PP2A via decreasing PP2A-A and PP2A-B expressions in AML cell lines and primary human AML blasts [22]. Agarwal et al. also indicated that primary AML cells inhibit PP2A by overexpressing SET [23]. At present, reactivation of PP2A is recognized as a potential therapy for FLT3-driven survival and proliferative signals in AML. For example, several PP2A-activating drugs (PADs), such as OP449 and FTY720 (a SET antagonist), were reported to enhance a cell death-inducing effect of FLT3 TKIs in FLT3-ITD-driven AML cell lines and primary AML cells [22,23]. In addition to the TKIs, PADs in combination with cytotoxic chemotherapy for treatment of various cancer types including AML have been widely explored. For example, pharmacological restoration of PP2A by FTY720 was reported to induce caspase-dependent apoptosis and AKT and ERK signaling inhibition in breast and colorectal cancers. The combination of FTY720 with doxorubicin and 5-fluorouracil can enhance the antitumor activities of these chemotherapeutic drugs against breast and colorectal cancers, respectively [41,42].

In AML with the NRAS mutation, OP449, was shown to increase Ara-C-induced cell death [23]. In addition to the FLT3 mutation, the NRAS mutation is another oncogenic mutation frequently observed in AML, and common downstream signaling pathways of both oncogenes that drive proliferation and survival of AML are phosphoinositide 3-kinase (PI3K)/Akt and MEK/ERK cascades [43]. Recently, SET upregulation was observed in AML cell lines and in primary AML patient samples harboring the FLT3-ITD and NRAS (Q61L) mutations [23]. Although whether EF-24 can prevent SET/PP2A-C binding needs to be further addressed, our current study showed that the PP2A inhibitor, OA, can reverse 2 µM EF-24-induced ERK deactivation, suggesting that high concentrations of EF-24 can suppress ERK activation via activating PP2A in HL-60 AML cells. Targeting the ERK pathway by EF-24 to induce apoptosis was also observed in oral cancer [44]. Therefore, EF-24 might be a novel PAD to target the ERK pathway, and combinations of EF-24 with chemotherapeutic agents or TKIs might have promising synergistic effects in treating AML.

In addition to ERK deactivation, a high concentration of EF-24 (2 µM) triggered p38 MAPK activation to induce caspase-mediated apoptosis. Our results were similar to previous reports, which indicated that p38 MAPK is involved in the caspase-mediated apoptotic effect caused by several natural products in AML cells [45]. Previous studies indicated that p38 MAPK activation-mediated inhibition of ERK activation provides a functional link between these two signaling pathways to regulate apoptosis [46]. We further investigated the relationship between ERK deactivation and p38 activation under EF-24 treatment and found that EF-24-induced dephosphorylation of ERK and cleavage of caspase-3 and PARP are all significantly reversed by the p38 inhibitor SB203580, suggesting the negative regulation of ERK activity by p38-induced apoptosis of HL-60 AML cells after treatment with a high concentration of EF-24 (2 µM). A previous report indicated that PP2A activation is critical for the negative regulation of p38 on ERK activity and apoptosis induction in cardiac myocytes [47]. In human skin fibroblasts, arsenite can induce p38-mediated PP2A activation to suppress ERK activity [48]. In human neutrophils, Avdi et al. reported p38 MAPK-dependent PP2A activation [49]. Our present study also showed that a p38 inhibitor reversed the EF-24 (2 µM)-induced activation of PP2A, suggesting that EF-24 might activate p38 to upregulate PP2A activity and further deactivate ERK in HL-60 AML cells. How p38 MAPK affects PP2A activation is still unknown and needs to be further investigated in the future.

The effects of EF-24 on MAPK and PP2A activities appear to be dependent on the cell type and concentration. Our present results showed that moderate concentrations of EF-24 (0.5 and 1 µM) specifically activated the ERK pathway and deactivated PP2A, whereas a high concentration (2 µM) seemed to preferentially activate p38 MAPK and PP2A. We observed that caspase-8/-3 activation and the decrease in the number of viable cells, triggered by a moderate concentration of EF-24 (1 µM), were enhanced by the ERK inhibitor, U0126. A moderate concentration of EF-24 (1 µM)-induced ERK activation was reversed by FTY720, suggesting that PP2A-regulated ERK activation might be a cell-derived protective effect caused by the toxic effects from moderate concentrations of EF-24 treatment. Similar results from previous studies also showed that an ERK inhibitor enhanced tricetin (a natural product) induced cell survival inhibition of AML cells [50]. Taken together, we propose that EF-24 combined with an ERK inhibitor may be a good antileukemic strategy for AML cells.

In addition to mild apoptosis induced by moderate concentration of EF-24 (1 μM), we observed that cell-cycle S phase arrest can be induced by the same concentrations of EF-24 (1 μM) in HL-60 and U937 cells (IC_50_ of EF-24: 0.8 and 0.9 μM). We also observed that autophagy-related marker Beclin-1 was induced at this concentration of EF-24 treatment (1 μM) in HL-60 cells (Figure S7, left panel); however, the apoptosis was dramatically induced at the high concentration of 2 μM. Similar to HL-60 cells, another autophagy-related marker LC3-II was induced by moderate concentration of EF-24 (0.5 μM) treatment in MV4-11 cells (IC_50_ of EF-24: 0.4 μM; Figure S7, right panel) and apoptosis was triggered in high concentration of EF-24 (1 μM). We observed that a broad-spectrum caspase inhibitor Z-VAD-FMK attenuated a 2 μM EF-24-induced decrease in the proportion of viable HL-60 cells, but had no significant effect on the 1 μM EF-24-induced decrease in the proportion of viable cells (Figure S8).

Previous reports indicated that CUR shows anticancer activity against oral, lung, and leukemic cancers via inducing both autophagy and apoptosis [51,52,53]. Similar to our results, Yu et al. indicated that IC_50_ of EF-24 on the viability of A549 lung cancer cells was 8.5 μM and a moderate concentration of EF-24 (8 μM) mainly induced autophagy, but a high concentration of EF-24 (16 μM) triggered cell apoptosis. They found that inhibition of autophagy can increase cell survival [54]. Li et al. also reported that CUR exerted an antiproliferative effect on HL-60 AML cells via inducing autophagy, apoptosis, and S-phase cell cycle arrest [55]. Taken together, we suggest that S-phase cell cycle arrest and autophagic cell death might be the main explanations for the reduction in the proportion of viable cells after moderate concentration of EF-24 treatment. Moreover, Z-VAD-FMK only partially reversed 2 μM EF-24-induced decrease in the proportion of viable cells from 84% to 69%, suggesting that a caspase-independent pathway is also involved in the cytotoxic effect of EF-24 in HL-60 AML cells.

## 4. Materials and Methods

### 4.1. Cell Lines

The MV4-11 human AML cell line was provided by Dr. L.-I. Lin (National Taiwan University, Taipei, Taiwan), whereas the U937 and HL-60 cell lines were purchased from the ATCC (Manassas, VA, USA). All cell lines were cultured in RPMI 1640 medium supplemented with 10% fetal bovine serum, 2 mM L-glutamine, 0.1 mM nonessential amino acids, 100 µg/mL streptomycin, and 100 U/mL penicillin.

### 4.2. Chemicals and Reagents

EF-24 and DMC (purity of ≥98%; HPLC), FTY720 (a PP2A activator), and ZVAD-FMK (a general inhibitor of caspases) were purchased from Sigma Chemical (St. Louis, MO, USA). Propidium iodide (PI) was obtained from Invitrogen (Carlsbad, CA, USA). U0126 (an ERK1/2 inhibitor), JNK-in-8, and SB203580 were purchased from Calbiochem (San Diego, CA, USA). The PP2A inhibitor, okadaic acid (OA), and an anti-β-actin antibody were obtained from Abcam (Cambridge, MA, USA). An antibody specific for the phosphorylated form of PP2A-Cα (Tyr307) was purchased from Thermo Fisher Scientific (Waltham, MA, USA). Antibodies against LC3B, pro-caspase-8/9, cleaved caspase-8/-9/-3, PARP, and unphosphorylated or phosphorylated forms of ERK1/2 and JNK1/2 were all obtained from Cell Signaling Technology (Danvers, MA, USA). Antibodies against pro-caspase-3, phospho-p38, and unphospho-p38 were purchased from BD Biosciences (San Jose, CA, USA). An antibody specific for beclin-1 was obtained from Santa Cruz Biotechnology (Santa Cruz, CA, USA).

### 4.3. Cytotoxicity Assay

The cytotoxic effect of EF-24 and DMC on AML cells was measured using Cell Counting Kit-8 (CCK-8; #96992 from Sigma Chemical). HL-60, U937, and MV4-11 cells were plated in a 24-well plate with 3 × 10^4^ cells/well. Then, cells were treated with indicated concentrations of EF-24 or DMC for 24 h and incubated of 10 μL of a CCK-8 solution with 100 μL of complete media containing 3 × 10^3^ cells in each well of a 96-well plate. The plate was next incubated at 37 °C for 4 h, and the absorbance was read at 450 nm by a microplate reader (MQX200; Bio-Tek Instruments, Winooski, VT, USA).

### 4.4. Fluorescence-Activated Cell Sorting Analysis of the Cell Cycle Distribution

AML cells (4 × 10^6^) were seeded in 6 cm dishes and treated with indicated concentrations of EF-24 (0, 0.125, 0.25, 0.5, 1, and 2 μM) for 24 h. After treatment, cells were washed with PBS and fixed with ice-cold 75% ethanol at −20 °C for 12 h. Cells were then incubated with 0.5 mL PI/RNase staining buffer for 15 min in the dark followed by filtration through a 40 μm nylon mesh (Falcon, San Jose, CA, USA). DNA contents of stained cells were detected by a BD Accuri C6 flow cytometer (BD Biosciences, San Jose, CA, USA) or Attune NxT flow cytometer (Thermo Fisher Scientific, Carlsbad, CA, USA) and analyzed by their accompanied software or FlowJo software.

### 4.5. Apoptosis Assays

AML cells (4 × 10^6^) were treated with EF-24 (0, 0.125, 0.25, 0.5, 1, and 2 μM) for 24 h. Apoptotic cell death induced by EF-24 was determined following the manufacturer’s guidelines of the FITC Annexin V Apoptosis Detection Kit I (no. 556547; BD Biosciences, San Jose, CA, USA).

### 4.6. Protein Lysate Preparation and Western Blot Analysis

The protein lysates preparation and the Western blot analysis followed previously described procedures [56].

### 4.7. Bioinformatics Analysis

Survival z-scores for *PPP2CA* gene in various cancer types including AML were obtained from PRECOG (PREdiction of Clinical Outcomes from Genomic profiles, Stanford, CA, USA) which is a publicly available, curated, and integrated meta-analysis of expression signatures from around 26,000 human tumors. This online resource includes overall survival (OS) outcomes, encompassing 166 cancer-expression datasets across 39 distinct malignancies, comprising 1261 cases of AML. PRECOG z-scores represent the number of standard deviations from the mean of a normal distribution and are directly related to *p*-values, encoding the directionality and robustness of statistical associations.

### 4.8. Statistical Analysis

Values are revealed as the mean ± SD. Statistical analyses were performed using SigmaPlot, vers. 10.0 (Systat Software Inc., San Jose, CA, USA). Differences were considered at *p*-values of <0.05.

## 5. Conclusions

**Figure 6:**
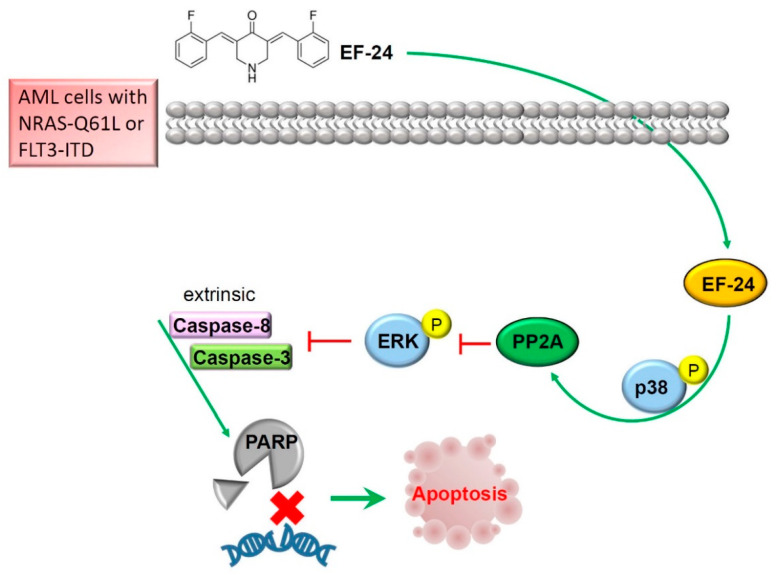
Schematic illustration of the molecular mechanism underlying the ability of EF-24 to trigger apoptotic cell death of acute myeloid leukemia (AML) cells. The antileukemic activity of EF-24 was attributed to turning on the activation of the p38 mitogen-activated protein kinase (MAPK)/protein phosphatase 2A (PP2A) axis, with subsequent attenuation of the extracellular signal-regulated kinase (ERK) activity and ultimate induction of extrinsic apoptotic cell death.

In conclusion, we first report that EF-24, a monoketone analog of CUR, exerts an antileukemic effect on AML cells. This phenomenon is caused by the induction of p38 MAPK activation, which initiates a signal leading to PP2A-mediated ERK activity loss and triggers extrinsic proapoptotic signaling. The mechanism is schematically illustrated in Figure 6. In the clinic, the PP2A expression level was correlated with more favorable outcomes in AML compared to other solid tumor types. This novel mechanism of EF-24 discovered in our study not only provides further insights into its antileukemic potential, but also contributes to developing EF-24 as a useful PP2A-activating agent for treating AML, especially in patients with the NRAS or FLT3 mutation.

## Supplementary Materials

The following are available online at https://www.mdpi.com/2072-6694/12/8/2163/s1. Figure S1: EF-24 induces an increase of the cell population in the sub-G1 phase.; Figure S2: Flow cytometric analysis of apoptotic human U937 acute myeloid leukemia (AML) cells stained with propidium iodide (PI) and FITC-Annexin V after treatment with EF-24.; Figure S3: EF-24 induces caspase-dependent apoptosis in MV4-11 acute myeloid leukemia (AML) cells.; Figure S4: EF-24 induces cell-cycle arrest at S phase.; Figure S5: Effect of EF-24 with different concentrations on dynamic changes of phosphatase 2A (PP2A), extracellular signal-regulated kinase (ERK)1/2, and p38 activities in MV4-11 acute myeloid leukemia (AML) cells.; Figure S6: EF-24 induces caspase-dependent apoptosis via activating phosphatase 2A (PP2A) in MV4-11 acute myeloid leukemia (AML) cells.; Figure S7: Effect of various concentrations of EF-24 on autophagy-related markers in acute myeloid leukemia (AML) cells.; Figure S8: High concentration of EF-24 induces caspase-dependent apoptosis in HL-60 acute myeloid leukemia (AML) cells. Figure S9: Whole Blots for Western Blot analysis for Figure 2C. Figure S10: Whole Blots for Western Blot analysis for Figure 2D. Figure S11: Whole Blots for Western Blot analysis for Figure 3A. Figure S12: Whole Blots for Western Blot analysis for Figure 3C. Figure S13: Whole Blots for Western Blot analysis for Figure 4A. Figure S14: Whole Blots for Western Blot analysis for Figure 4D. Figure S15: Whole Blots for Western Blot analysis for Figure 5A. Figure S16: Whole Blots for Western Blot analysis for Figure 5C. Figure S17: Whole Blots for Western Blot analysis for Figure 5D. Figure S18: Whole Blots for Western Blot analysis for Figure 5E. Figure S19: Whole Blots for Western Blot analysis for Figure S3. Figure S20: Whole Blots for Western Blot analysis for Figure S5. Figure S21: Whole Blots for Western Blot analysis for Figure S6. Figure S22: Whole Blots for Western Blot analysis for Figure S7.
