# Supplementary material for: The Curcumin Analogue, EF-24, Triggers p38 MAPK-Mediated Apoptotic Cell Death via Inducing PP2A-Modulated ERK Deactivation in Human Acute Myeloid Leukemia Cells
Source: Cancers (Basel). 2020 Aug 4;12(8):2163. doi: 10.3390/cancers12082163 (PMC7464750; doi:10.3390/cancers12082163)
Supplement: Supplementary file 1 [file cancers-12-02163-s001.pdf]

cancers

MDPI

Supplementary Materials

The Curcumin Analogue, EF-24, Triggers p38 MAPK-Mediated Apoptotic Cell Death via Inducing PP2A-Modulated ERK Deactivation in Human Acute Myeloid Leukemia Cells

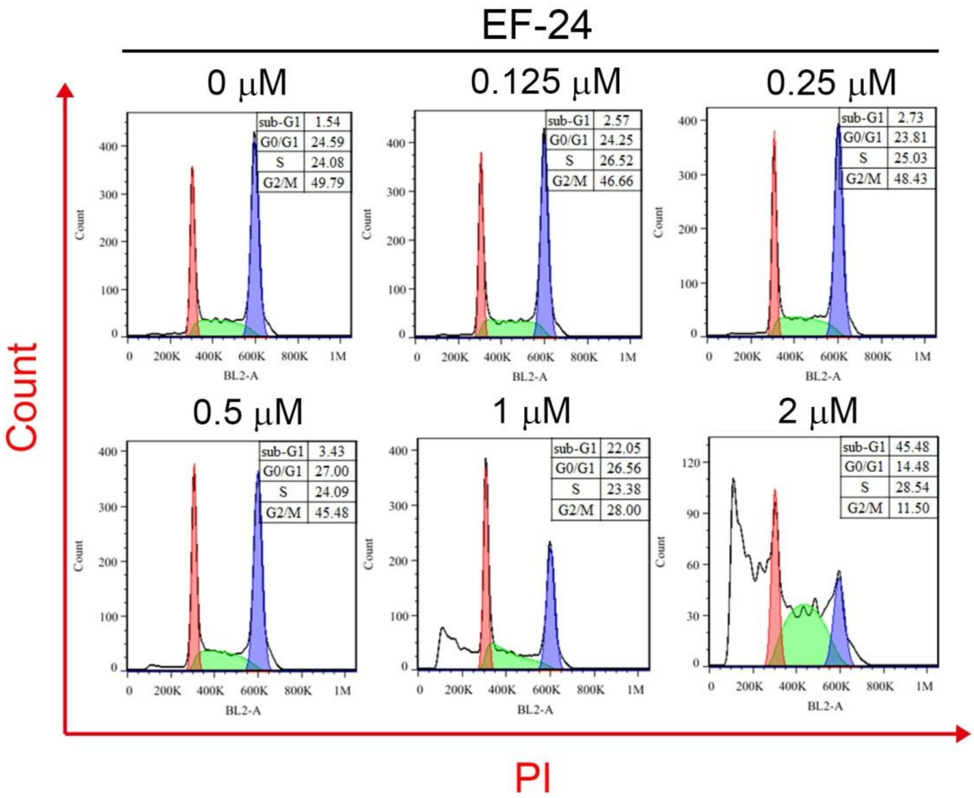

Figure S1. EF-24 induces an increase of the cell population in the sub-G_1 phase. Treatment of U937 cells with indicated concentrations of EF-24 for 24 h. The cell-cycle phase distribution and cell death in the sub-G_1 phase were analyzed by FACS after propidium iodide (PI) staining. Data are shown as the cell-cycle distribution profile by FACS, and percentage distributions of cells in the sub-G_1, G_0/G_1, S, and G_2/M phases are inserted in the graphs.

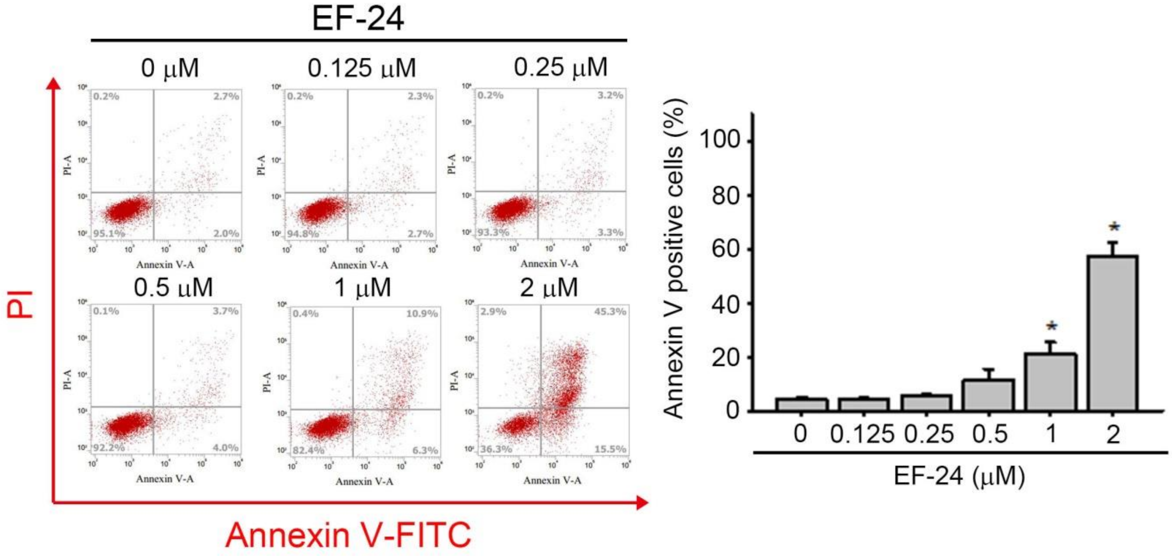

Figure S2. Flow cytometric analysis of apoptotic human U937 acute myeloid leukemia (AML) cells stained with propidium iodide (PI) and FITC-Annexin V after treatment with EF-24. AML cells were treated with EF-24 at indicated concentrations and its vehicle (0.5% DMSO) for 24 h. Then, numbers of cells in four fractions (PI-negative/FITC-Annexin V-negative, PI-positive/FITC-Annexin V-negative, PI-negative/FITC-Annexin V-positive, and PI-positive/FITC-Annexin V-positive) were counted, and their distribution was analyzed. (A) Representative flow cytometric dot plots. (B) A summary of populations of U937 cells stained with only FITC-Annexin V (early apoptosis) and with both FITC-Annexin V and PI (late apoptosis). Data are shown as the mean ± standard deviation (SD) (n = 3). * $p < 0.05$ vs. vehicle treatment.

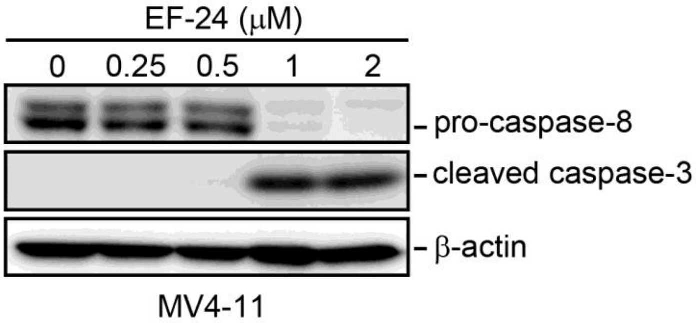

Figure S3. EF-24 induces caspase-dependent apoptosis in MV4-11 acute myeloid leukemia (AML) cells. Cells were treated with EF-24 at indicated concentrations and its vehicle (0.5% DMSO) for 24 h, and levels of pro-caspase-8 and cleaved caspase-3 were analyzed by a Western blot analysis. Whole Blots for Western Blot analysis for Figure S3 are shown in the Figure S19.

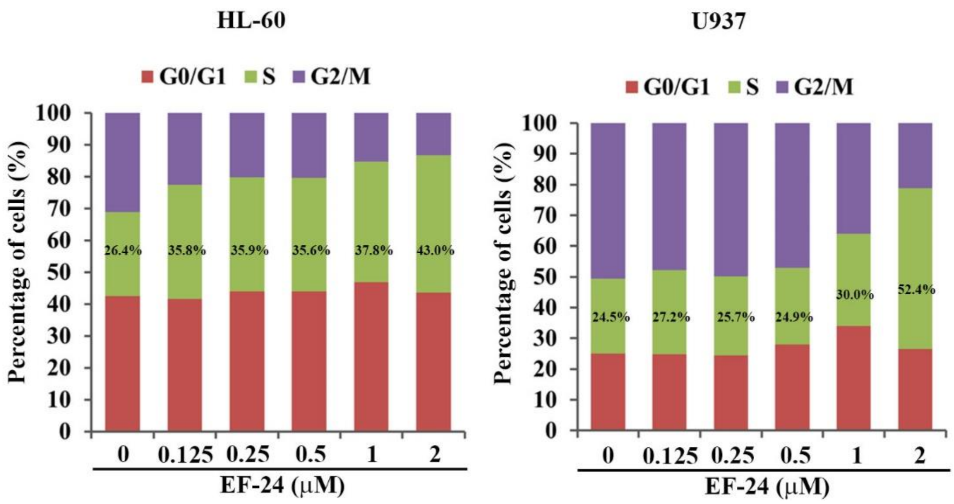

Figure S4. EF-24 induces cell-cycle arrest at S phase. Treatment of HL-60 and U937 cells with indicated concentrations of EF-24 for 24 h. The cell-cycle phase distribution was analyzed by FACS after propidium iodide (PI) staining. The percentage distributions of cells in the S phase are inserted in the graphs.

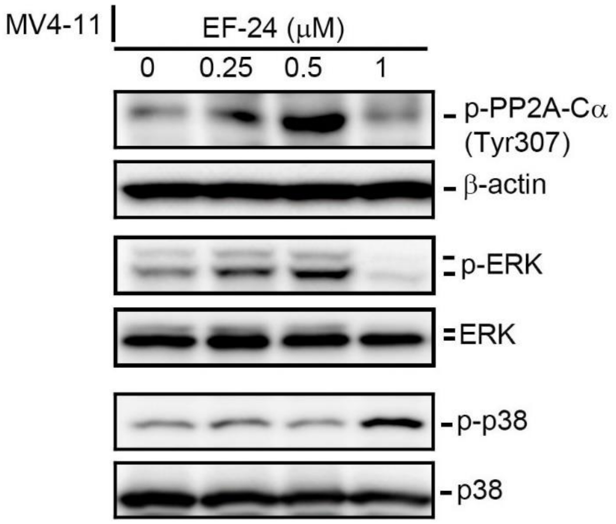

Figure S5. Effect of EF-24 with different concentrations on dynamic changes of phosphatase 2A (PP2A), extracellular signal-regulated kinase (ERK)1/2, and p38 activities in MV4-11 acute myeloid leukemia (AML) cells. After 24h treatment of MV4-11 cells with indicated concentrations (0.25, 0.5, and 1 μM) of EF-24, levels of phosphorylated ERK1/2, p38, and PP2A-Cα were detected by a Western blot analysis. Whole Blots for Western Blot analysis for Figure S5 are shown in the Figure S20.

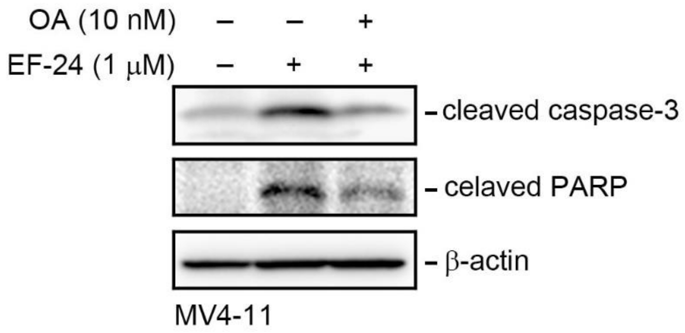

Figure S6. EF-24 induces caspase-dependent apoptosis via activating phosphatase 2A (PP2A) in MV4-11 acute myeloid leukemia (AML) cells. MV4-11 cells were pretreated with or without 10 nM okadaic acid (OA) for 1 h followed by EF-24 (1 μM) treatment for another 24 h. A Western blot analysis was used to determine the expression levels of cleaved caspases-3 and poly(ADP-ribose) polymerase (PARP). Whole Blots for Western Blot analysis for Figure S6 are shown in the Figure S21.

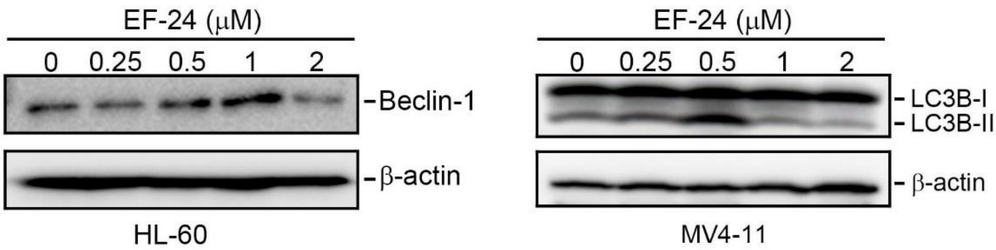

Figure S7. Effect of various concentrations of EF-24 on autophagy-related markers in acute myeloid leukemia (AML) cells. HL-60 and MV4-11 cells were treated with EF-24 at the indicated concentrations for 24 h, and Beclin-1 or light chain 3 (LC3)-II expression was detected by a Western blot analysis. Whole Blots for Western Blot analysis for Figure S7 are shown in the Figure S22.

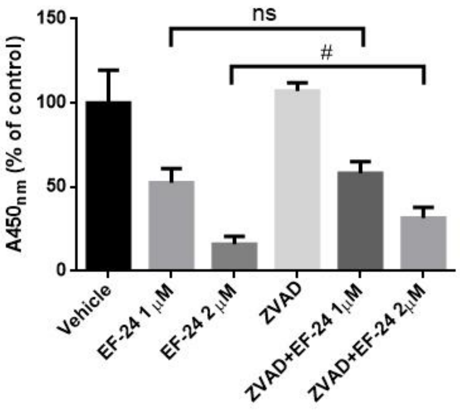

Figure S8. High concentration of EF-24 induces caspase-dependent apoptosis in HL-60 acute myeloid leukemia (AML) cells. Cells were treated with moderate (1 μM) or high (2 μM) concentration of EF-24 for 24 h in the presence or absence of 20 μM Z-VAD-FMK. The proportion of viable cells was determined by a CCK8 assay. Values are presented as the mean ± standard deviation (SD) of three independent experiments. $^{\#}$ $p < 0.05$, EF-24 (2 μM) vs. Z-VAD plus EF-24. ns: not significant.

Whole Blots for Western Blot analysis

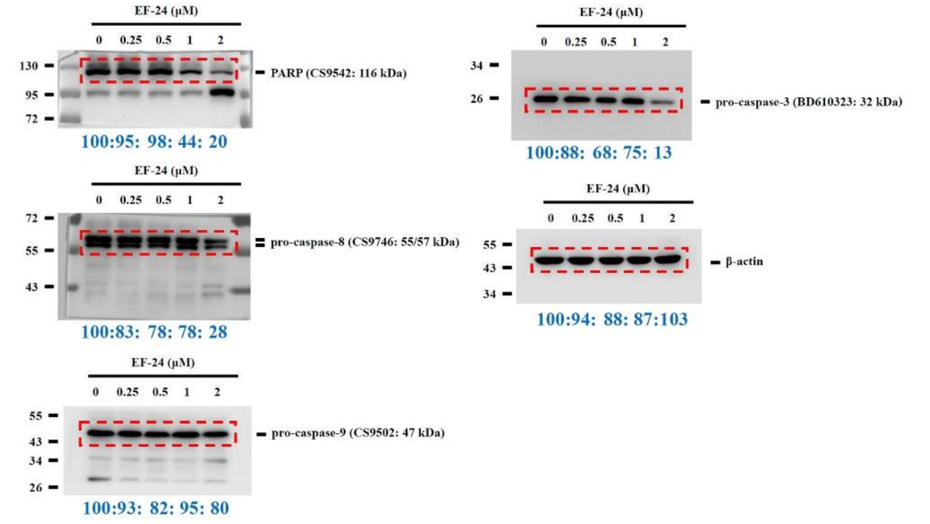

Figure S9. Whole Blots for Western Blot analysis for Figure 2C.

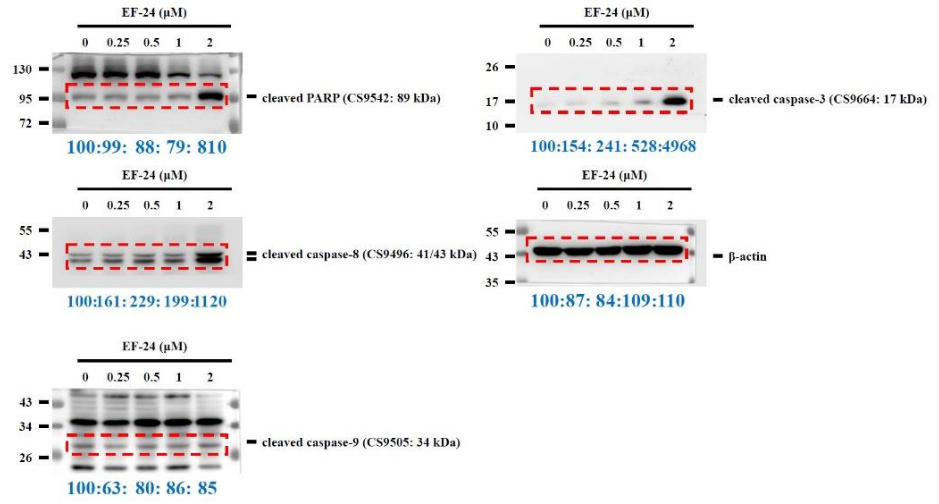

Figure S10. Whole Blots for Western Blot analysis for Figure 2D.

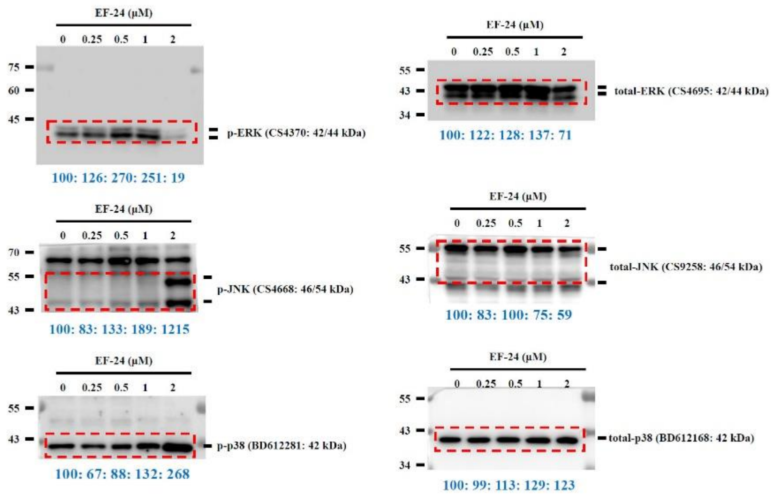

Figure S11. Whole Blots for Western Blot analysis for Figure 3A.

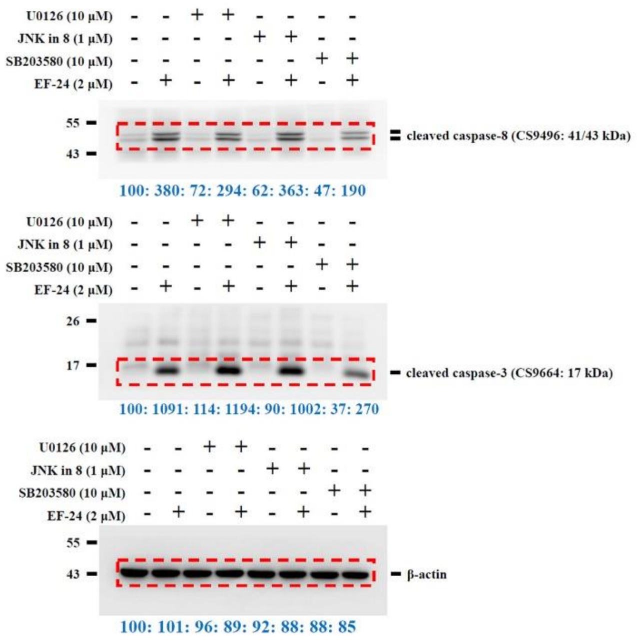

Figure S12. Whole Blots for Western Blot analysis for Figure 3C.

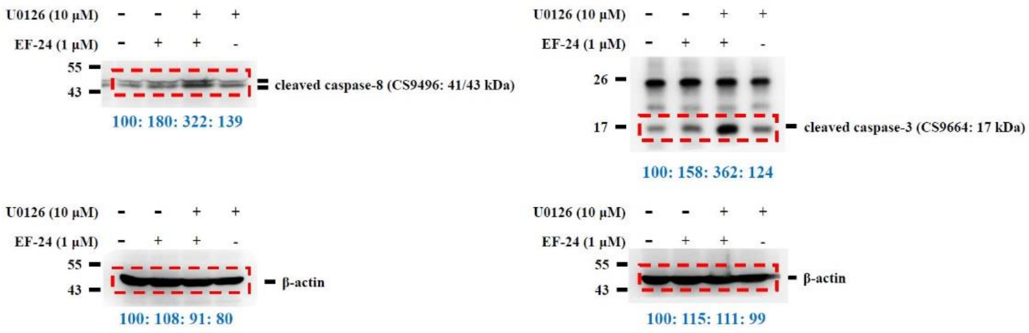

Figure S13. Whole Blots for Western Blot analysis for Figure 4A.

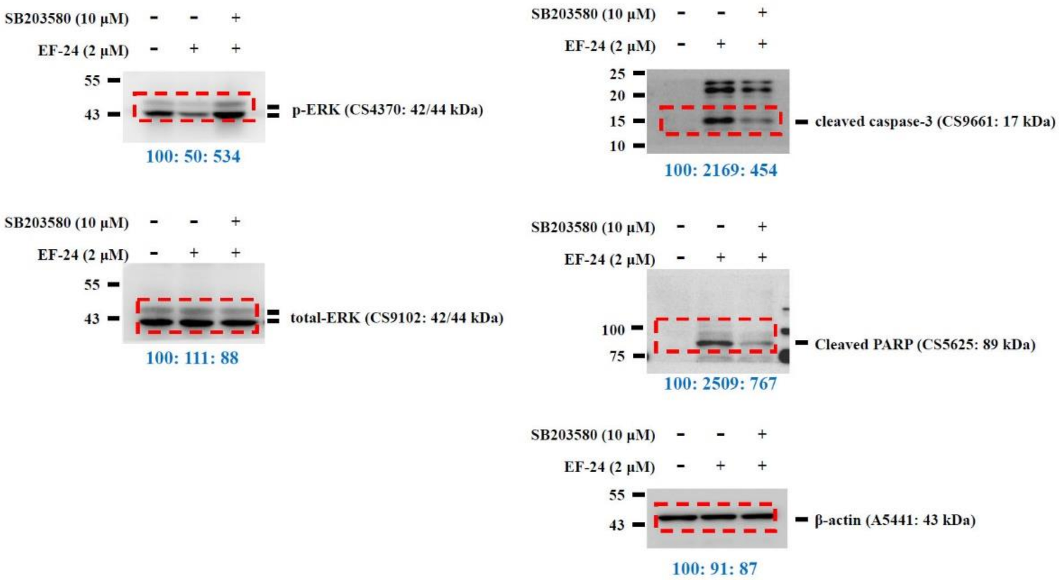

Figure S14. Whole Blots for Western Blot analysis for Figure 4D.

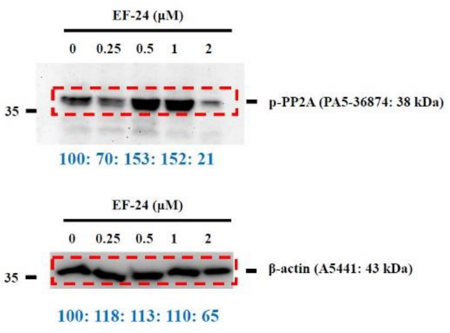

Figure S15. Whole Blots for Western Blot analysis for Figure 5A.

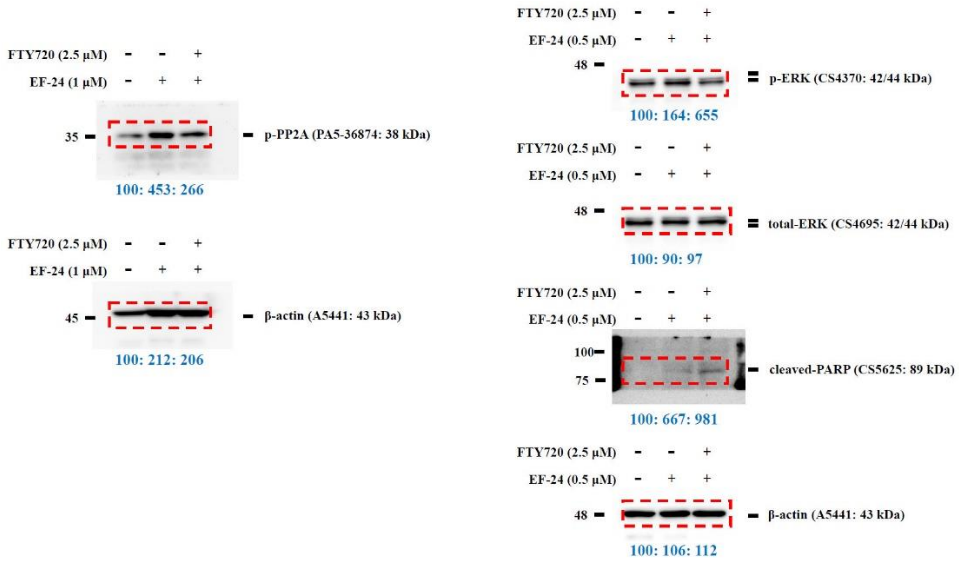

Figure S16. Whole Blots for Western Blot analysis for Figure 5C.

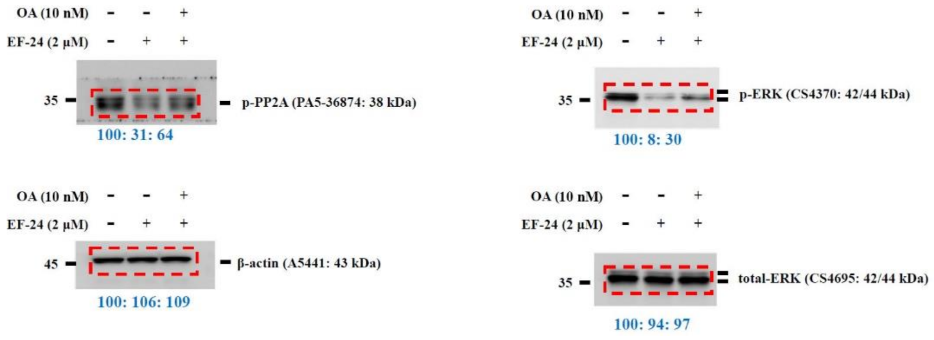

Figure S17. Whole Blots for Western Blot analysis for Figure 5D.

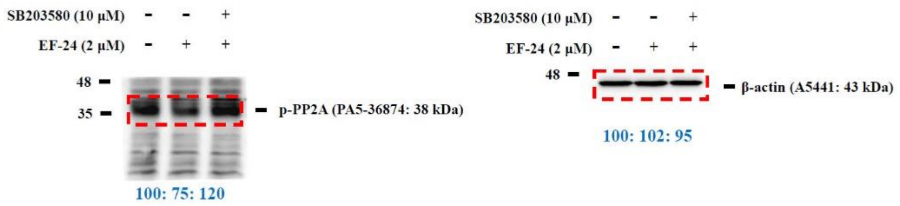

Figure S18. Whole Blots for Western Blot analysis for Figure 5E.

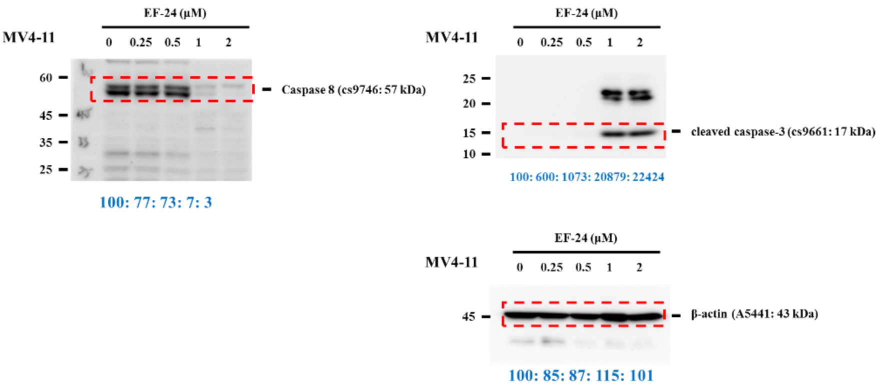

Figure S19. Whole Blots for Western Blot analysis for Figure S3.

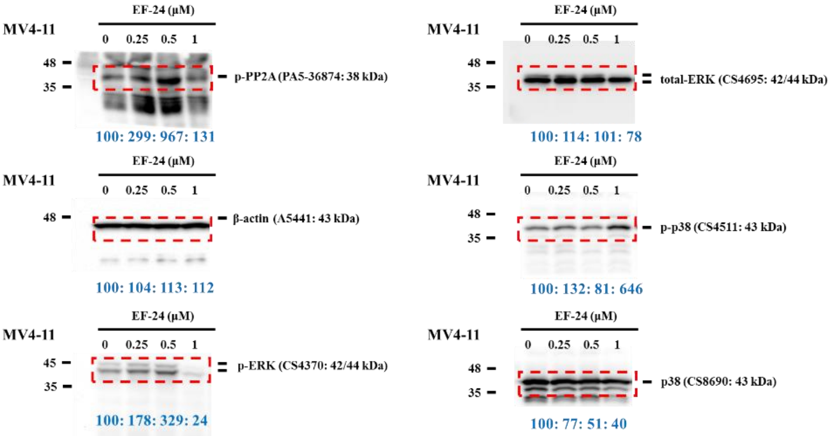

Figure S20. Whole Blots for Western Blot analysis for Figure S5.

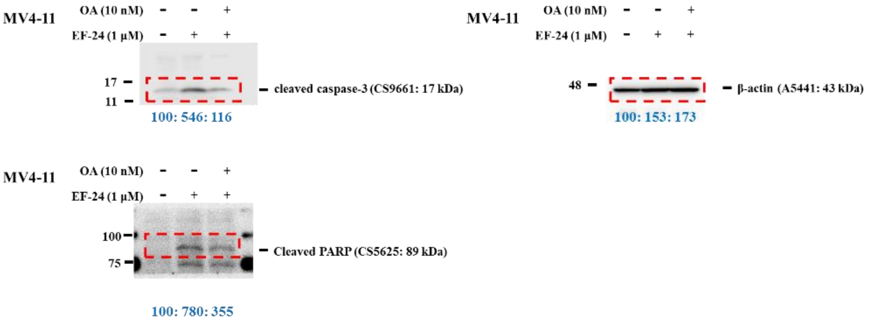

Figure S21. Whole Blots for Western Blot analysis for Figure S6.

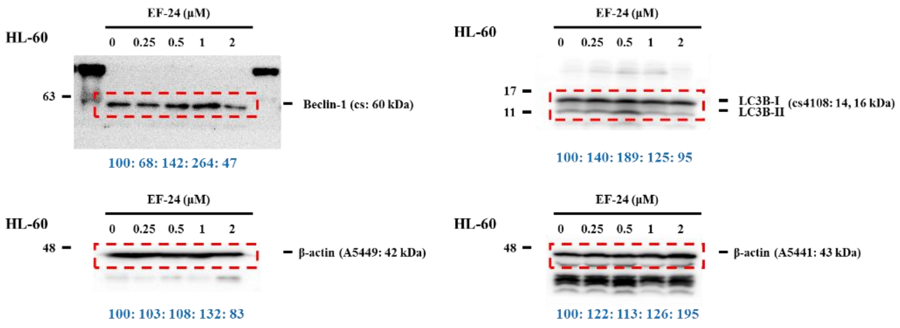

Figure S22. Whole Blots for Western Blot analysis for Figure S7.

© 2020 by the authors. Licensee MDPI, Basel, Switzerland. This article is an open access article distributed under the terms and conditions of the Creative Commons Attribution (CC BY) license (http://creativecommons.org/licenses/by/4.0/).
